# Repurposing Drugs by* In Silico* Methods to Target BCR Kinase Domain in Chronic Myeloid Leukemia

**DOI:** 10.31557/APJCP.2019.20.11.3399

**Published:** 2019

**Authors:** Aparna Natarajan, Rajkumar Thangarajan, Sabitha Kesavan

**Affiliations:** *Department of Molecular Oncology, Cancer Institute (WIA), Adyar, Chennai, India. *

**Keywords:** BCR, ABL, TKI’s, Grb-2, Tyrosine 177, computational drug repurposing, docking studies

## Abstract

**Background:**

Targeted therapy in the form of highly selective tyrosine kinase inhibitors (TKIs) has transformed the treatment of chronic myeloid leukemia (CML). However, mutations in the kinase domain contribute to drug resistance against TKIs which compromises the treatment response. Our aim is to explore regions outside the *BCR-ABL* oncoprotein to identify potential therapeutic targets to curb drug resistance by targeting growth factor receptor-bound protein-2 (Grb-2) which binds to *BCR-ABL* at the phosphorylated tyrosine (Y177) thereby activating the Ras and PI3K/AKT signaling pathway.

**Methods:**

We have used in silico methods to repurpose drugs for identifying their potential to inhibit the binding of Grb-2 with Y177 by occupying the active binding site of the *BCR* domain.

**Results:**

Differentially expressed genes from GEO dataset were found to be associated with hematopoietic cell lineage, NK cell-mediated cytotoxicity, NF-κB and chemokine signaling, cytokine-cytokine receptor interaction, histidine metabolism and transcriptional misregulation in cancer. The fold recognition method of SPARKS-X tool was used to model the *BCR *domain (Z-score = 8.21). Connectivity Map generated a drug list based on the gene expression profile, which were docked with *BCR*. Schrodinger XP glide docking identified Diphosphopyridine nucleotide, Hesperidin, Butirosin, Ovoflavin, and Nor-dihydroguaiaretic acid to show strong interaction in close proximity to the active binding pocket containing Y177 of the target protein and was further validated using iGEMDOCK and Parallelized Open Babel and AutoDock suite Pipeline (POAP).

**Conclusion:**

Our study not only extends our current knowledge about repurposing drugs for newer indications but also provides a route towards combinatorial therapy with standard drugs used for CML treatment. However, the efficacy of these repurposed drugs needs to be further investigated using in vitro and in vivo studies.

## Introduction

Chronic myeloid leukemia (CML) is a clonal myeloproliferative disorder characterized by the presence of a balanced reciprocal translocation between the breakpoint cluster region (*BCR*) gene on chromosome 22q11.2 and the Abelson gene (*ABL1*) on chromosome 9q34, resulting in the formation of t(9;22)(q34;q11) (Di Bacco et al., 2000). The *BCR-ABL* chimeric oncogenic protein constitutively activates tyrosine kinase, driving leukemic cells through the phosphorylation of downstream effector molecules such as Grb2, RAK, ROS, PI3K, JNK, STAT5, AKT and Myc, which in turn activate numerous signal transduction pathways and lead to uncontrolled cell proliferation (Deininger et al., 2000; Pendergast et al., 1993). The incidence of CML is about 1-2 cases per 100,000 adults and accounts for approximately 15% of the newly diagnosed cases of leukemia (Jabbour and Kantarjian, 2016). 

The blast cell percentage divides CML into asymptomatic chronic phase (CP), accelerated phase (AP) and blast crisis (BC) with the accumulation of additional genetic abnormalities as the disease inevitably progresses to a more aggressive form. Alterations in the *p53* and *Rb1* genes occur in about 30% and 20% of blast crisis cases, respectively, and are responsible for the clonal evolution. About 20% of the patients may transit directly to BC without evolving into AP and become poor responders with a failure rate of >70% (Di Bacco et al., 2000; Jabbour and Kantarjian, 2016; Jain et al., 2017). 

CML is the first human malignancy for which targeted therapy was applied. The frontline treatment using the FDA approved first-generation tyrosine kinase inhibitor Imatinib mesylate to block the cellular proliferation of the malignant clones drastically reduced the annual mortality rate to 1-2% and improved the long-term overall survival rates to >80-90% (de Kogel and Schellens, 2007; Mauro and Druker, 2001; Facts, 2015; Deininger et al., 2009; Hess et al., 2008; Hochhaus et al., 2008, 2017; Palandri et al., 2008).

The BCR-ABL protein contains multiple functional domains and motifs capable of transforming the primitive hematopoietic cells into malignant leukemic cells by disrupting the regulation of many signaling pathways and cellular functions. The currently practiced targeted therapy fails to eliminate the complete population of CML progenitor cells having ABL kinase domain mutation that leads to imatinib resistance; hence, our interest is to explore the targets outside this domain. The first exon on the* BCR* domain contains tyrosine 177 (Y177), which activates the transforming potential of the cells and synergistically promotes CML cell expansion, proliferation and survival. The autophosphorylated Y177 of *BCR-ABL* recruits an adapter protein Grb2 (growth factor receptor-bound protein 2) and binds the SH2/SH3 domain with high affinity. The *BCR-ABL-Grb-2 *interaction leads to Grb2-SOS complex formation, which triggers the downstream activation of PI-3K-AKT through SOS mediated Ras-MAPK signaling (Chu et al., 2000; Zhang et al., 2001). Y177 also recruits the scaffolding adaptor Gab2 via a Grb2/Gab2 complex for effective induction of the myeloproliferative disease (Sattler et al., 2002). Mutations involving Y177 prevent Grb2 binding, thereby decreasing the signaling and hence the proliferation of Ph+ myeloid progenitor cells (Million and Van Etten, 2000; Pendergast et al., 1993). 

Drug development is time-consuming and expensive with extremely low success and relatively high attrition rates (Wu et al., 2013). Despite the enormous investment ranging from 500 million USD to 2 billion USD, the number of drugs being approved has been declining since the late 1990s (Boguski et al., 2009). Recycling existing licensed drugs for new medical indications gave the benefits of reducing the development cost and time as well as the probability of failure by exploiting the readily available pharmacokinetic properties, adverse effects/ toxicities, evidence from clinical trials, and post-marketing safety data (Deotarse et al., 2015). Protein structure predictions and molecular docking studies are reliable ways of screening thousands of drugs interacting with specific targets to shortlist a set of prospective FDA approved compounds to be repositioned for off-label uses (Candidate and Stratagies, 2018; Xue et al., 2018). 

A notable example is Thalidomide, a sedative initially used to treat leprosy, which was then repurposed for multiple myeloma, once it was identified to inhibit the angiogenesis induced by fibroblast and vascular endothelial growth factors (Richardson et al., 2002). Likewise, anti-cancer drugs such as Imatinib (Demetri et al., 2002), Sorafenib (Zhang et al., 2008), Crizotinib (Carpenter and Mosse, 2012), Gemcitabine (Zhang et al., 2017) and Ponatinib (Musumeci et al., 2018) are being successfully employed to treat various cancers apart from their original indication owing to their ability to target multiple pathways. Computational methods are also being used to identify new ways of tackling the *BCR-ABL* mutations by devising novel inhibitors that target the mutated clones (Banavath et al., 2014). We now propose to repurpose drugs identified by in silico methods to effectively target the serine/threonine (S/T) kinase domain of BCR, thereby blocking the Grb2 binding which leads to inhibition and progression of CML.

## Materials and Methods

*Identification of differentially expressed genes*

Dataset GSE33075 (Benito et al., 2012) was downloaded from Gene Expression Omnibus (GEO) (Barrett et al., 2007). The dataset contained nine healthy bone marrow samples (donor) and hematopoietic cells from 9 Ph+ CML patients at diagnosis and their corresponding samples after one month of therapy with 400 mg of OD imatinib. The data were analyzed by categorizing the samples into defined groups: donor with untreated CML and pre vs. post imatinib treatment. GEO2R, an interactive web tool provided by NCBI (version R 3.2.3, Biobase 2.30.0, GEOquery 2.40.0, Limma 3.26.8), was used to perform the calculations (Edgar, Domrachev, and Lash, 2002). 

*Target pathway identification*

The obtained differentially regulated genes were used as input for the “Database for Annotation, Visualization and Integrated Discovery” (DAVID v6.8) to establish the gene ontology (GO terms) and analyze the KEGG pathway (Huang, Sherman, and Lempicki, 2009b, 2009a).

*Connectivity Map (CMap)*

The drugs associated with the differentially expressed genes were ascertained based on their phenotypic expression profile. CMap is a comprehensive drug perturbation database containing 6,100 data points derived from genome-wide transcriptional expression data using 3,000 chemical compounds in three cultivated cancer cell lines, thereby serving as a reference database (Ravindranath et al., 2015). The drugs at the top of the list are strongly correlated and those at the bottom are strongly anti-correlated functionally based on the query state through the transitory feature of common gene expression changes (Musa et al., 2018; Wang et al., 2018).

*BCR interacting partners*

The STRING database provides the networks and functional associations between proteins on a global scale. It predicts the protein-protein interactions between our target protein BCR and its interacting partners based on direct (physical) and indirect (functional) associations (http://string-db.org) (Szklarczyk et al., 2015), which have been provided with a probabilistic confidence score based on the selected value. 

*Modeling and Docking*

*Model building*

The amino acid sequence of the BCR protein (kinase domain) was retrieved from UniProt (P11274), and its structure was predicted using the SPARKS–X tool (http://sparks.informatics.iupui.edu/) (Yang et al., 2011). If the sequence identity was <40%, the domains and its chains were included in the library for model generation with modeler9v7 (Sali et al., 1995) using the alignment produced by SPARKS-X. The different models were linked, steric clashes were removed using the DFIRE potential functions (Yang et al.,, 2008; Zhou and Zhou, 2002), and the protein model was generated. 

*Protein preparation and validation*

The model which gave a Z-score >8 in the SPARKS-X tool was selected for further validation. The PDB file of the BCR kinase domain was processed using the “Protein Preparation Wizard” of Schrodinger suite 2018-1. All the missing hydrogen atoms were added and the orientation of side chains containing the glutamine, asparagine, and histidine residues was prepared. The protonation states of histidine were assigned, and energy minimization was performed with the OPLS force field (Sastry et al., 2013). The protein was evaluated using the RAMPAGE Ramachandran plot analysis (Lovell et al., 2003).

*Active site prediction*

The active site of the BCR kinase domain was recognized using the SiteMap tool (Halgren, 2007; Halgren, 2009). The binding site having a score >1 was selected for further docking studies. 

*Ligand preparation*

The drugs attained from the CMap tool were corrected using the Ligprep tool (Schodinger Release 2018-1: LigPrep, Schodinger, LLC, New York, NY, 2018). The molecules were converted to their 3D structures, and their geometries were optimized by the addition of hydrogen. 

*Receptor grid generation*

Using the receptor grid generation module of Schrodinger, the grid was created at the active site of the amino acids (identified by SiteMap), that is, the Grb2 binding region of the BCR kinase domain, by means of default parameters. 

*Docking*

*Schrodinger 2018-1 suite*

Glide docking was carried out with the prepared ligands using the extra precision (XP) scoring (Friesner et al., 2004, 2006; Halgren et al., 2004) function. The compounds were ranked based on the scores, and the specific interactions such as hydrogen bonding, Pi – cation, and Pi -Pi between the protein and ligands were visualized using ligand interaction tools.

*iGEMDOCK*

iGEMDOCK is a graphical-automatic drug design system for docking, screening, and post-analysis, which calculates the energy of each pose and generates its fitness by calculating the individual energy terms (Yang and Chen, 2004; Yang and Shen, 2005). 

*Parallelized Open Babel and AutoDock suite Pipeline (POAP)*

POAP integrates the tools such as Open Babel, AutoDock, AutoDock Vina and AutoDockZN in an easily configurable Bash shell-based text interface. The modules for ligand preparation such as single receptor virtual screening, multiple receptor virtual screening, and consensus scoring were performed. POAP calculates the ligand binding energy and scoring based on the AutoDock Lamarckian Genetic Algorithm and free energy empirical scoring (Morris et al., 2009; Samdani and Vetrivel, 2018). The energies obtained from POAP were compared with the ligand score identified from Schrodinger. 

## Results

*Identification of gene expression changes between the donors and CML patients*

The differentially expressed genes between the donors and CML patients at diagnosis (Set A) and those between the pre and post imatinib treated patients (Set B) showing fold changes >2 were identified. There were 92 up-regulated and 250 down-regulated genes in Set A. In Set B, 262 up-regulated and 183 down-regulated genes were obtained. All gene lists are shown in Supplementary Table S1 (A and B). The genes present in the two sets were compared using InteractiveVenn (Heberle et al., 2015) (Supplementary Figure 1a and 1b). The common genes acted in opposing ways, that is, up-regulated in Set A but down-regulated in Set B and vise-versa, implying that they could be probable targets for drug identification. (Supplementary Table S2)

*Characterizing the differentially expressed genes with DAVID by using Gene Ontology and KEGG pathway analysis*

Gene ontology was performed using DAVID for the genes from Set A and B to infer the functional consequences of the up-regulated and down-regulated genes. The significant cutoff of the EASE score, a modified Fisher Exact test value (p-value) of 0.01, was set to identify the GO-terms. The differentially expressed genes were also mapped using the Kyoto Encyclopedia of Genes and Genomes (KEGG) pathway. It was found that the hematopoietic cell lineage, natural killer cell-mediated cytotoxicity, NF-κB signaling pathway, chemokine signaling pathway, transcriptional misregulation in cancer, and histidine metabolism were involved in Set A. On the other hand, chemokine signaling, cytokine-cytokine receptor interaction, viral carcinogenesis, and Fc gamma R-mediated phagocytosis were involved in Set B. The complete list of GO ontology terms and KEGG pathway are given in Supplementary Table S3 (A and B).

*Candidate drugs from CMap*

The perturbations from CMap yielded detailed results from the gene signatures analyzed according to their permutated results, P-values and enrichment scores. The robust connection between the genes and the drugs was applied to obtain candidates for docking. In Set A, 1,156 drugs gave a positive score and 1,648 drugs gave a negative score. On the other hand, in Set B 1,052 and 493 drugs showed positive and negative scores respectively. The complete list of drugs are given in Supplementary Table S4 (A and B). 

*STRING protein-protein interactions*

**Figure 1:**
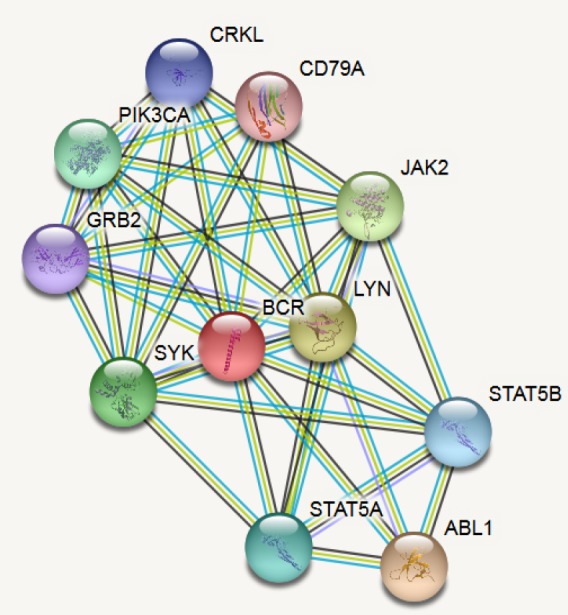
BCR Interacting Partners from STRING database

The STRING database was used to identify if BCR interacts with Grb-2 such that it can be targeted to inhibit the proliferation of the leukemic clones. To recognize the specific and meaningful interactions, the minimum required interaction score was set at the highest confidence of 0.900. The active interaction sources were from text mining, databases, and co-expression. A maximum of 10 interactions were allowed to detect the top-scoring partners. BCR interacts with Grb2 with a score of 0.958. The interacting partners are represented in Figure 1.

*Protein structure prediction and validation*

The BCR gene (UniPort ID: P11274) consists of 1,271 amino acids, of which the first 426 belong to the serine-threonine kinase domain. The SPARKS-X tool generated 10 models based on the Z-score using different templates, amongst which the one with the highest score value of 8.21 was selected for validation. (Supplementary data S5)

**Figure 2.**
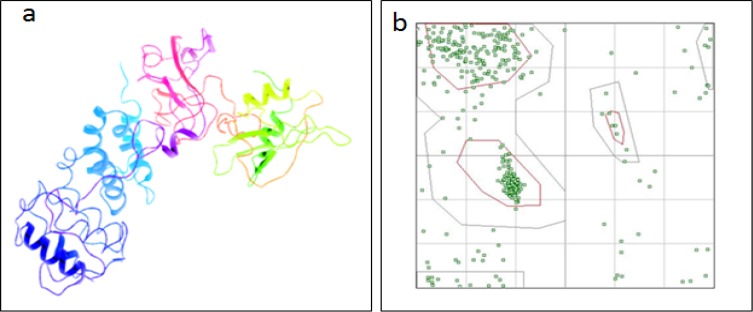
Protein Modeling and Validation. (a) BCR protein structure from SPARK-X tool (b) Ramachandran plot

The protein preparation wizard of Schrodinger was used to correct the modeled BCR protein structure for virtual screening. The bond orders were assigned, and the hydrogen atoms were added to optimize the structure. Restrained minimization with a fixed RMSD of 0.3Å using the OPLS3 force field was performed. The minimized structure thus obtained is depicted in Figure 2a. Furthermore, the structure was validated using the RAMPAGE software, which indicated 84.4% as residues in the favored region, 9.7% as those in the allowed region, and 5.9% as outliers. The Ramachandran plot generated is given in Figure 2b. The validated structure was then used for docking studies. The active binding pocket with a site score of 1.058 was chosen, whose residues are as follows: 130-140, 158, 177-180, 182, 184, 186-192, 195, 200-203. Tyrosine 177 was one of the various residues in the active binding pocket, which was used to generate the grid using a grid generation panel. 

*Docking results*

**Figure 3:**
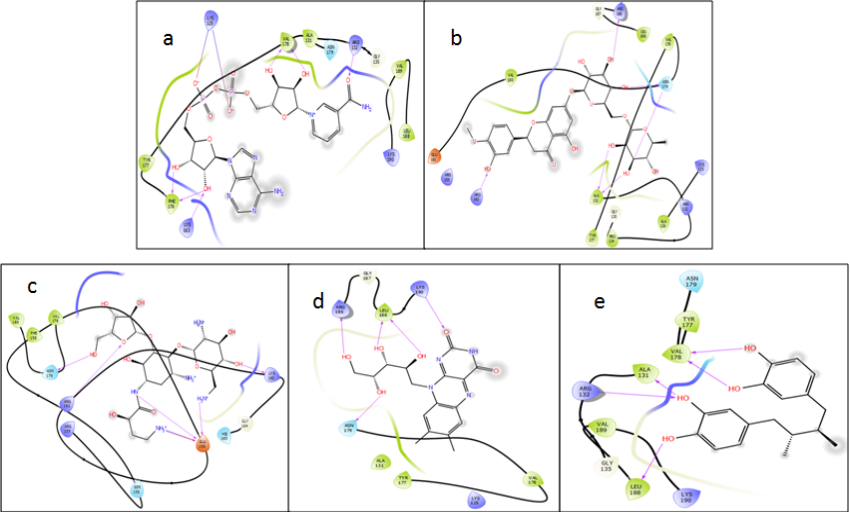
Ligand Interactions Using Schrodinger XP. (a), Diphosphopyridine nucleotide; (b), Hesperidin; (c), Butirosin; (d), Ovoflavin; (e), Nordihydroguaiaretic acid

The docking score revealed the list of compounds that can form hydrogen bonds with the active binding region of the BCR protein, thereby increasing the chance of being repurposed for a therapeutic effect. A total of 32 compounds (Supplementary Table S6) were discerned, including antibiotics, antimicrobials, flavonoids, and contrast agents. Five candidates, namely nadide (NAD+), hesperidin, butirosin, ovoflavin and nordihydroguaiaretic acid (NDGA), displayed strong interactions in close proximity to the active binding pocket containing Y177 of the BCR protein (Figure 3). Table 1 lists the various residues that interact with the drugs to be repurposed for CML.

**Table 1:** Glide XP Results for the Ligand Interactions, by Schrodinger and Validation Using iGEMDOCK and POAP

Chembl Id	Compound (Drug Bank)	Interacting Residues	Xp G Score	Glide Energy	iGEMDOCK	Poap
1234613	Nadide	Lys125,Arg132,Lys163,Phe176, Val178	-8.257	-61.723	-214	-9
449317	Hesperidin	Ala131,Arg162,Val178,Asn179Arg186	-6.739	-41.925	-179.7	-8.8
376923	Butirosin	Glu159,Arg162,Lys163,Asn179	-6.901	-46.131	-225.3	-5.9
511565	Ovoflavin*	Asn179,Arg186,Leu188,Lys190	-5.755	-32.374	-208.6	-7
52	Nordihydro-guaiaretic acid	Ala131,Arg132,Val178,Leu188	-5.652	-33.604	-184.3	-8.4

*Drug names obtained from Pubchem

## Discussion

This work aims at repurposing well-known formulations to identify the connections between the molecular biology of CML and its treatment prospects in patients with progressive or resistant disease. The potential drugs capable of inhibiting the condition were identified by in silico molecular docking based on the XP glide score and glide energy. Among the top 32 candidates selected for repurposing, Deferoxamine, Mitoxantrone, and Leucovorin were also present which have already been identified for cancer treatment. Recent research published by Yujing et al., (2018) has revealed the protective role of Deferoxamine in leukemic cell apoptosis by regulating the concerned genes. Iron chelators have also been reported to exert anti-proliferative effects in various cancers, including head and neck (Donneys et al., 2018; Donneys et al., 2018), colorectal (Cao et al., 2018) and breast (Bajbouj et al., 2018; Tury et al., 2018). Mitoxantrone is a well-known antitumor antibiotic used for the treatment of relapsed acute lymphoblastic leukemia (ALL) (Parker et al., 2010), acute myeloid leukemia (AML), breast cancer, non-Hodgkin’s lymphoma, and hormone therapy failed advanced prostate cancer (Shenkenberg and Von Hoff, 1986). Leucovorin, an active metabolite of folic acid, is being used as an anti-cancer enhancer along with fluorouracil (Poon et al., 1989) or as a chemoprotectant along with methotrexate (Levitt et al., 1973). Such evidences prove that in silico docking can be used as the first step to screen thousands of known compounds to identify ideal drug candidates for repositioning. 

NAD+ plays a crucial role in diverse cellular processes. The targets from NAD+ metabolic pathways, such as rate-limiting enzyme NMPRTase, Indoleamine 2.3-dioxygenase (IDO) and Inosine mononucleotide dehydrogenases (IMPDH), have been reviewed for their anti-cancer effects (Khan et al., 2007). Studies reveal that NAD+ acts as a protective factor in early carcinogenesis as it prevents or restores the malignant phenotype of cancer cells by inducing cellular repair, stress adaptive response, regulation of cell cycle arrest and apoptosis (Poljsak, 2016). The coenzyme has also been observed to exert significant anti-cancer effects by blocking proliferation and inducing apoptosis in B-cell ALL (Takao et al., 2018) and chronic lymphocytic leukemia (CLL) (Audrito et al., 2011). 

Hesperidin is a promising natural bioflavonoid that has been investigated for its anti-cancer property in various solid tumors including gastric, colon, breast, lung and liver. The compound induces apoptosis in cancer cells through NF-κβ, p53, PPAR-γ, PI3K/AKT and mTOR signaling pathways (Pandima et al., 2015). Besides, the flavonoid exerts cytotoxic and proapoptotic effects on ALL cells by blocking the PI3K/Akt pathway and inhibiting NF-κB activation (Shahbazi et al., 2018). 

Butirosin is a water-soluble amino cyclitol glycosidic antibiotic complex known to be active against many gram-positive and some gram-negative bacteria. Existing data assert that antibiotics can affect the kinase signaling pathways and the secretion of cytokines which are known to promote cancer stem cell expansion. The drug has been proven to reduce the viability and clonal expansion of breast cancer stem cells (Pestell and Rizvanov, 2015). Butirosin was one among the 20 small molecules identified to reverse prostate cancer by in silico method. CMap analysis revealed an enrichment score of -0.821, indicating that the drug can be exploited as an adjuvant to improve the therapeutic effect for prostate cancer (Wen et al., 2014). Similarly, latent pathway identification analysis (LPIA) and pathway–pathway interactions showed that butirosin and neomycin biosynthesis (hsa00524) significantly interact with other pathways. The identified genes serve as attractive targets for intervention to enhance the prognosis in pediatric ALL (Gao et al., 2015). 

Ovoflavin is a member of vitamin B complex, which is usually taken as a supplement. The vitamin is found in cells and tissues chiefly as flavin mononucleotide (FMN) and flavin adenine dinucleotide (FAD). Of late, vitamins are being explored for their role in the prevention and treatment of cancer. Vitamin B_2_ sensitizes breast and lung cancer cell lines to vitamin C in a synergistic way by inducing cell death through the inhibition of Akt and Bad phosphorylation (Chen et al., 2015). 

NDGA functions as an anti-tumorigenic and anti-proliferative agent in various cancers, including those of the breast, prostate, lung, esophageal and skin (Lu et al., 2010). The 5-lipoxygenase inhibitor, NDGA, selectively inhibits the expression of cyclin D1 in pancreatic and cervical cancer cells. This process is accompanied by the activation of Jun-NH2-terminal kinase and p38 mitogen-activated protein kinase, inducing anoikis-like apoptosis. Disruption of the actin cytoskeleton in association with the activation of stress-activated protein kinases has also been observed (Seufferlein et al., 2002). NDGA, along with the extract from the fungus *Lecanicillum lecanii,* inhibits the growth of lymphatic leukemia cells by inducing nuclear damage leading to reduced DNA content in the cells (Bibikova et al., 2017). Besides, the compound causes cell death in AML cell lines as well as in patient samples via inhibition of AKT phosphorylation, suggesting that the extrinsic and the mitochondrial apoptotic pathways are not essential for cell death (Mak et al., 2007). 

Previous work performed in our lab using synovial sarcoma cell lines containing SYT-SSXI fusion protein has revealed that the compounds identified by in silico methods have promising anti-proliferative effects. Target gene expression studies confirmed the down-regulation of the fusion protein, and cell cycle analysis showed enhanced apoptotic cell death (Natarajan et al., 2018). Thus, we conclude that the proposed set of drugs can be reinvestigated for their potential role in CML treatment. These compounds can be used either as part of a combination therapy or to reduce the adverse effects of chemotherapy along with the existing treatment regimens as they are fairly non-toxic and inexpensive. However, the efficacy of these drugs needs further evaluation.
